# Early Motor Differences in Infants at Elevated Likelihood of Autism Spectrum Disorder and/or Attention Deficit Hyperactivity Disorder

**DOI:** 10.1007/s10803-020-04489-1

**Published:** 2020-04-23

**Authors:** Jannath Begum Ali, Tony Charman, Mark H. Johnson, Emily J. H. Jones, and the BASIS/STAARS Team

**Affiliations:** 1 Centre for Brain and Cognitive Development, Department of Psychological Sciences, Birkbeck, University of London, Henry Wellcome Building, Malet Street, London, WC1E 7HX UK; 2 Psychology Department, Institute of Psychiatry, Psychology & Neuroscience, King’s College London, London, UK; 3 Department of Psychology, University of Cambridge, Cambridge, UK

**Keywords:** Autism spectrum disorder, Attention deficit hyperactivity disorder, Reaching, Motor ability, Midline crossing

## Abstract

We investigated infant’s manual motor behaviour; specifically behaviours crossing the body midline. Infants at elevated likelihood of Autism Spectrum Disorder (ASD) and/or Attention Deficit Hyperactivity Disorder (ADHD) produced fewer manual behaviours that cross the midline compared to infants with a typical likelihood of developing these disorders; however this effect was limited to 10-month-olds and not apparent at age 5 and 14 months. Although, midline crossing did not predict ASD traits, it was related to ADHD traits at 2 years of age. We rule out motor ability and hand dominance as possible explanations for this pattern of behaviour, positing that these results may be a consequence of multisensory integration abilities, and the neurobehavioural shift period, in the first year of life.

**Electronic supplementary material:**

The online version of this article (10.1007/s10803-020-04489-1) contains supplementary material, which is available to authorized users.

## Introduction

Transdiagnostic approaches to neurodevelopmental disorders have gained increasing traction over the last decade (e.g., Livingston and Happé 2017; Rigney et al. 2018; Peralta and Cuesta 2017). Such approaches recognise growing evidence that the aetiology of a range of mental health conditions significantly overlap, both in terms of genetic (e.g., Meda et al. 2014; Owen and O’Donovan 2017) and environmental (e.g., Beauchaine and Constantino 2017; Chisholm et al. 2015) risk factors. Attempts to dissect relevant dimensional domains include the Research Domain Criteria Framework (Insel et al. 2010), which has been suggested as an alternative to clinical diagnostic frameworks in ‘carving nature at its joints’. However, one challenge to transdiagnostic approaches is applying them to clinical cohorts. Such cohorts are recruited on the basis of discrete clinical diagnoses, and this may create significant confounds in our ability to identify shared versus distinct phenotypes. Further, a central question is the developmental emergence of convergence or divergence in early risk profiles, which requires longitudinal studies before the age at which clinical diagnosis can be made. One solution to this issue is to examine transdiagnostic phenotypes in longitudinal studies of populations enriched for subsequent development of a range of neurodevelopmental disorders.

Two conditions in which a transdiagnostic approach might be particularly appropriate are Autism Spectrum Disorder (ASD) and Attention Deficit Hyperactivity Disorder (ADHD). Both are early-onset neurodevelopmental disorders that can be diagnosed by early to mid-childhood. Both significantly co-occur, with 20–50% of children diagnosed with ASD also meeting the diagnostic criteria for ADHD (Russell et al. 2014; Stevens et al. 2016). Further, the two conditions are associated with the same range of co-occurring conditions such as anxiety (Shephard et al. 2018; Gordon-Lipkin et al. 2018), depression (Davidsson et al. 2017), cognitive impairment (Corbett et al. 2009), and motor problems (Reiersen et al. 2008; Piek and Dyck 2004). However, the two conditions can be readily and reliably distinguished in clinical assessments (Mayes et al. 2012). In a recent review, Johnson et al. (2015) propose that understanding the nature of the overlap between ASD and ADHD requires longitudinal prospective studies that trace the developmental trajectories of risk and protective factors for the two conditions. Taking such an approach allows us to identify the mechanisms that contribute to both phenotypic overlap and divergence.

In the present study, we focus on early motor function as a potentially transdiagnostic phenotype that is critical in early development, and has been implicated in both ASD and ADHD. ASD is a neurodevelopmental disorder characterised by social communication difficulties, restrictive and repetitive behaviours and sensory anomalies (DSM-5, 2013). Whilst not included in diagnostic frameworks, the high prevalence of motor difficulties has led some to argue that this is a core feature of the disorder (Fournier et al. 2010; Leary and Hill 1996). Indeed, research has identified differences within oral-motor functioning, postural control, co-ordination, manual control, gross and fine motor skills and reaching motor milestones (e.g., Gernsbacher et al. 2008; Noterdaeme et al. 2002; Iverson and Wozniak 2007; Minshew et al. 2004; Athanasiadou et al. 2019). Similarly, though ADHD is diagnosed through parent and caregiver report of inattention and hyperactivity (DSM-5, 2013), there is a high prevalence of motor difficulties (Kaiser et al. 2015). Domains affected include gross and fine motor skills, motor co-ordination, slower reaction times and preparation and execution of motor actions (Fliers et al. 2008; Klimkeit et al. 2005; see Kaiser et al. 2015 for a meta analysis). Thus, motor difficulties may be a common feature of both ASD and ADHD. However, the degree to which motor problems are a secondary consequence of behavioural symptoms of the two conditions (phenotypic causality and convergence) or a primary feature that emerges early in development and has cascading effects on both phenotypes is unclear.

There are several reasons to focus on motor skills in early development. Motoric skills allow children to explore their environment, gating self-directed experience (e.g., Adolph and Robinson 2015). Further, in typical development, motor ability has been tightly coupled with cognitive abilities such as language, memory and visual processing (Iverson 2010; Davis et al. 2011; Stöckel and Hughes 2015), with research indicating that improvements in perceptual skills (such as visual discrimination, working memory and selective attention) occurs as a by-product of motor training programmes (Alesi et al. 2015; Palter et al. 2011). The relationship between motor and cognitive skills has also been illustrated in conditions where there is significant motor impairment, specifically cerebral palsy. Individuals with cerebral palsy demonstrate delays in acquiring motor skills and reaching motor milestones (e.g., Allen and Alexander 1997) which can then impact cognitive functioning. For example, if they are unable to crawl or walk, this limits the environment in which they can actively explore to the space immediately around themselves, reducing the number of new sources of information about the world (Piaget 1952; Gibson 1988).

Further to this, delays in meeting motor milestones have also been implicated in conditions where motor impairment is not a core feature of the symptoms of the disorder, for example in schizophrenia. In a longitudinal study that followed individuals from the first year of life until 46–48 years of age, it was found that those participants that went on to a diagnosis of schizophrenia attained developmental motor milestones much later than individuals who did not go on to be diagnosed with the disorder (Sørensen et al. 2010). Additionally, gross motor deficits have been demonstrated in children and adolescents at an elevated likelihood of schizophrenia, by virtue of having a parent with the disorder, who then go on to obtain a diagnosis in adulthood (Erlenmeyer-Kimling et al. 2000). Interestingly, this study also demonstrated the predictive value of childhood verbal memory and attentional deficits for later diagnosis, which may lend itself to the tight coupling between motor and perceptual abilities.

Indeed, longitudinal studies have shown relationships between early motor delays and other domains of functioning, suggesting there may be cascading effects on other domains of development (Bedford et al. 2016; Leonard et al. 2015; Libertus and Violi 2016; Bhat et al. 2012; Flanagan et al. 2012). Motor skills are also important because they provide an early assessment of the integrity of the nervous system. Motor skills such as reaching, crawling and walking emerge gradually over the first years of life through a process of experience-dependent learning (see Adolph and Joh 2007; Thelen 1995). Closely tracking the development of such motor skills can thus provide a sensitive measure of a brain that is not developing optimally.

One particularly important early motoric achievement is goal-directed reaching. Reaching is defined as a manual behaviour whereby individuals manipulate the posture of their hand and arm in order to grasp an object. Infants do not engage in skilled reaching behaviours until just before 5 months of age (e.g., White et al. 1964; Galloway 2004; Thelen et al. 1993; von Hofsten 1984). Skilled reaching facilitates infants’ haptic and visual exploration of their environments during a period in which other major motor behaviours such as sitting up, crawling and walking are yet to develop. Accordingly, the development of skilled reaching has been linked to improvements in perceptual abilities like selective attention and action perception (Eppler 1995; Ruff and Rothbart 1996; Libertus and Needham 2010; Melzer et al. 2012).

Research examining reaching abilities using the infant sibling design has shown a number of atypicalities within this population (Libertus et al. 2014; Bryson et al. 2007; Ekberg et al. 2016). However, one question that remains is whether these atypicalities are specific to ASD or more general across a number of neurodevelopmental disorders.

A behaviour that emerges from skilled reaching is that of midline crossing. Midline crossing refers to the act of placing one’s hand in the contralateral side of space, thus crossing the body midline. When the ipsilateral arm is restrained, reaches that involve crossing the midline seem to emerge between 4 and 7 months (Provine and Westerman 1979). In contrast, spontaneous midline crosses (where the ipsilateral arm is not restrained) emerge a little later in development, at 6 months (Morange and Bloch 1996; White et al. 1964), as a consequence of bimanual reaching (van Hof et al. 2002). Thus, midline crossing emerges in the first half year of life, with the frequency of crossing increasing with age (Fagard et al. 2009; Melzer et al. 2012; Carlier et al. 2006; Pryde et al. 2000; Stilwell 1987). Crossing the midline is an important developmental milestone because it increases the type of interactions an infant can have with its environment as the right/left hand is no longer limited to the right/left sides of space. This then enables bimanual co-ordination and manipulation of objects, further increasing manual skill repertoire and opportunities for environmental interactions (Provine and Westerman 1979). Complementary arguments have proposed that midline crossing behaviours are also related to maturation of neuronal connections in the brain (specifically the corpus callosum) and hemispheric specialisation (Provine and Westerman 1979; Morange and Bloch 1996; Bishop 1990). Thus, examining the development of midline crossing and its relation to risk for ASD and ADHD can provide insight into critical mechanisms of experience-dependent learning that may form part of a phenotypic cascade to one or both disorders. Whilst there is, as yet, no research examining midline crossing behaviours in the infant sibling literature, there have been a few studies that have investigated this in older participants with a diagnosis of ASD. For example, Lagasse and Hardy (2013) describe a case study of ‘Mark’; a 7-year-old boy with ASD that has difficulties with motor co-ordination that included reaching across the midline without a tactile cue or prompt.

It is also important to consider whether midline crossing behaviours in early life are related to later emerging phenotypic characteristics of ASD and/or ADHD beyond infancy (e.g., in toddlerhood and early childhood). For example, it may be possible that midline crossings in the first year of life could potentially predict later ASD/ADHD traits at 2 years of life.

The current study examined the emergence and development of spontaneous midline crossing behaviours in infants with a first degree relative (older sibling or parent) with ASD and/or ADHD. Infants with an older sibling with ASD have an approximately 20% chance of developing ASD themselves (Ozonoff et al. 2011; see Jones et al. 2014). Recurrence rates of ADHD in similar designs have been less well characterised, but estimates of heritability are similar for both conditions (see Wade et al. 2015; Musser et al. 2014; Miller et al. 2019). We tested whether increased familial likelihood of ASD and/or ADHD influences the development of midline crossing behaviours in a naturalistic reaching task. To do this, we examined the ways in which infants at 5, 10 and 14 months of age engaged in manual behaviours when playing with toy blocks. Given the current landscape of research in motor impairments and midline crossing, we expect midline crossing behaviours will increase with age and there will be differences across the different participant groups. As the current literature is not entirely clear, it is difficult to make predictions regarding directionality of these differences. As such, we conducted two tailed tests on our dataset. To our knowledge, this is the first longitudinal study (in infancy generally) to investigate the developmental trajectory of midline crossing behaviours in a completely naturalistic task.

## Methods

### Stimuli

**Fig. 1:**
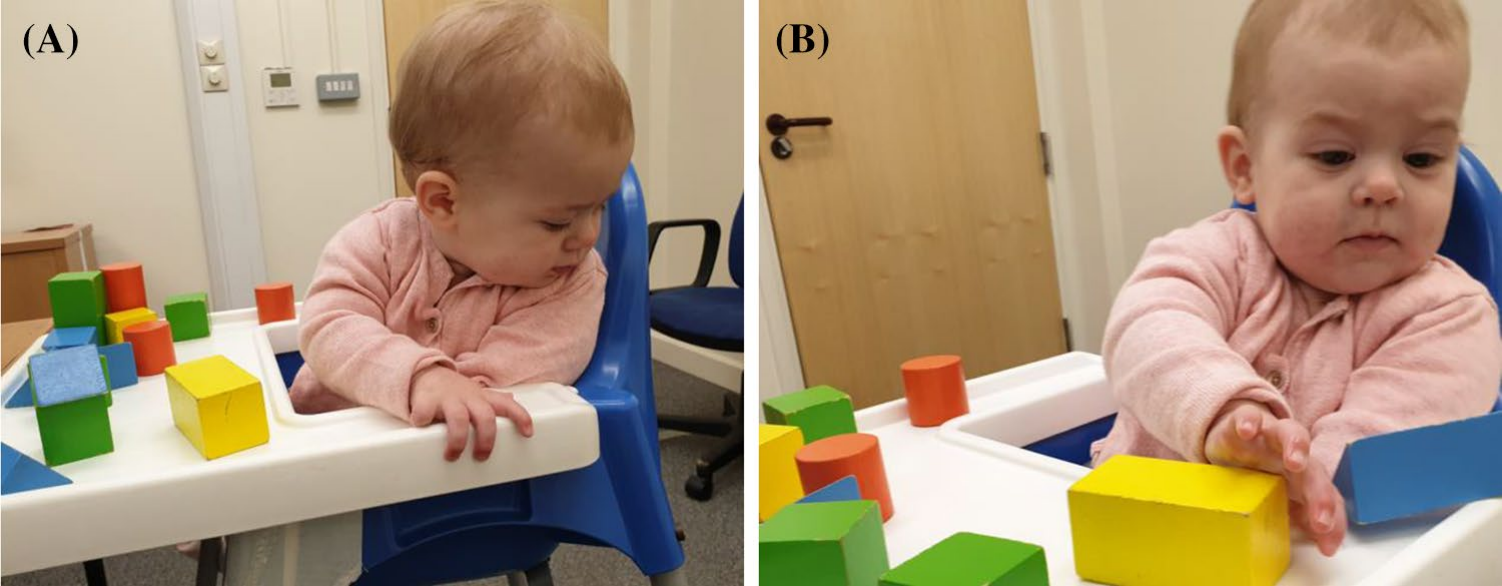
Photo of an infant completing the Blocks task. **a** ‘contralateral hand movement’ and **b** ‘contralateral reach’

There was a standard administration for this task. Infants were seated in either a baby high chair (10 and 14 month protocol) or on the caregiver’s lap, with the caregiver holding their trunk (5 month protocol). A box of foam (5 month protocol) or wooden (10 and 14 month protocol) blocks were placed on the table top in front of the infants. The blocks were always placed within reach of the infants, if they were ever positioned out of reach, either the researcher or the parent would place them back within reach. Three custom built cameras recorded infant’s behaviours (see Fig. 1). All infants were presented with the blocks for up to three minutes. Parents were instructed to allow their infant to play independently with the blocks, but not to intervene unless the infant became particularly fussy. If necessary, the researcher and/or parent encouraged the infant to play with the blocks, but parents did not play with the infant during the task.

### Participants

Participants were recruited for a longitudinal study running from 2013 to 2019. Infants could be enrolled in the study if they either had a first degree relative with ASD, a first degree relative with diagnosed or probable ADHD, or no first degree relatives with either diagnosis. We defined the presence of ASD as a clinical diagnosis of ASD from a licensed clinician.

We defined the presence of ADHD as a community clinical diagnosis of ADHD or a probable research diagnosis of ADHD. For those who report concerns of ADHD traits in the family where the parent or older sibling does not have a community clinical diagnosis of ADHD, screening questionnaires are used to examine the probable existence of ADHD. This was implemented because co-occurring conditions are often underdiagnosed in children with ASD (e.g., Musser et al. 2014; see Visser et al. 2016 for a review of co-occurrence rates) both because previously DSM-IV and ICD-10 gave primacy to an ASD diagnosis and due to lower rates of ADHD in children and adults compared to prevalence estimates (Sayal et al. 2018; Ginsberg et al. 2014) and, for example, to rates in the US (e.g., Merten et al. 2017). Had we required a clinical diagnosis for an infant to be coded as ‘elevated likelihood of ADHD, we would have risked under-identification in those families with a proband with an ASD diagnosis, significantly compromising the familial diagnosis elevated likelihood design we adopted for sampling. Further, we didn’t want to apply different criteria to those families with and without an older sibling with ASD. Thus, we adopted an additional screening process for ADHD in first degree relatives. For siblings (aged less than 6 years), a shortened version of the Conners Early Childhood (Conners 2008; Conners and Goldstein 2009) form is used. For siblings (6 years or older), a shortened version of the Conners 3 was used. Thresholds for inclusion were the presence of 6 ADHD traits on either the hyperactivity/impulsivity or inattention scale, and a positive score on the impairment scale. For parents a shortened version of the Conners Adults ADHD Rating Scale (CAARS) was used. Thresholds for inclusion were the presence of 5 ADHD traits on either the hyperactivity/impulsivity or inattention scale as per updated DSM V guidelines (see Table 1 for categorisation of the cohort). In terms of the use impairment scores, we used a reduced version of the Conners EC and Conners 3 for individuals under 18 and the CAARS for individuals aged 18 + years. The Conners EC and Conners 3 included questions regarding impairment, as such we also included these questions in our screening forms. In comparison, the CAARS (adult questionnaire) did not include questions regarding impairment. In order to maintain consistency of measure, we did not adapt the CAARS to add impairment questions. Of note, at initial contact with participants, parents were asked if there were any diagnoses of ADHD in the immediate family or if they had any concerns about ADHD. It is only if parents reported concerns that the screening process took place. This is a very similar categorisation protocol to that adopted by other labs using the prospective longitudinal study model in infants at elevated likelihood of ADHD (see Miller et al. 2020).

**Table 1:** Categorisation of the elevated likelihood cohorts

	EL-ASD	EL-ADHD	EL-ASD + ADHD
Parent reported diagnosis in older sibling	75	7	15
Parent reported diagnosis in parent	3	18	2
Parent reported diagnosis in both older sibling + parent	3	1	1
Screened parent (for ADHD traits)		4	1
Screened older sibling (for ADHD traits)		1	1

We defined no familial likelihood of ASD or ADHD as infants who had at least one older sibling with typical development and no first-degree relatives with a diagnosis of ASD or ADHD. These infants were recruited from a volunteer database at the Centre for Brain and Cognitive Development, Birkbeck University of London. Inclusion criteria included full-term birth (gestational age greater than 36 weeks). At the time of enrolment, none of the infants had a known medical or developmental condition. Informed written consent was provided by the parent(s) prior to the commencement of the study. The testing only took place if the infants were in a content and alert state. Ethical approval was granted by the National Research Ethics Service and the Research Ethics Committee of the Department of Psychological Sciences, Birkbeck, University of London. Participant families were reimbursed expenses for travel, subsistence and overnight stay if required. Infants were given a certificate and t-shirt after each visit.

Each infant in the study was assigned a rating for familial likelihood of ASD and ADHD. A rating of 1 for ASD indicates the presence of ASD in a parent or older sibling; a rating of 1 for ADHD indicates the presence of ADHD in a parent or older sibling; and a rating of 0 for either category indicates no *confirmed* presence of the relevant condition. Ratings were primarily based on the presence of a clinical diagnosis of ASD or ADHD. This approach allowed us to test the effect of familial likelihood of ASD, familial likelihood of ADHD, and their interaction (see Supplementary Materials Participants section for full details on this approach).

Our sample for this paper includes: 81 infants with an elevated likelihood of ASD (ASD-L), 31 infants with an elevated likelihood of ADHD (ADHD-L), 20 infants with an elevated likelihood of both ASD and ADHD (ASD + ADHD-L) and 29 infants with a Typical Likelihood for either ASD or ADHD (TL). A number of participants were excluded from the final analyses for fussy behaviour (n = 5 at the 5-month timepoint) or technical difficulties with the video recording (n = 4, n = 1 and n = 1 at the 5, 10 and 14 month time points respectively). See Table 2 for the final demographic breakdown by group at each time point.

**Table 2:** Participant characteristics of all participants included in the final analyses, across likelihood and age groups

	TL	EL-ASD	EL-ADHD	EL-ASD + ADHD
5 months				
*n*	26	52	16	13
Gender	17m, 9f	26m, 26f	9m, 7f	8m, 5f
Age in days (SD)	177.42 (13.69)	174.65 (20.18)	170.44 (15.57)	176.62 (14.87)
10 months				
* n*	27	77	26	20
Gender	16m, 11f	39m, 38f	15m, 11f	12m, 8f
Age in days (SD)	321.93 (16.7)	319.53 (14.84)	323.54 (27.75)	319.7 (14.66)
14 months				
*n*	23	73	25	19
Gender	13m, 10f	38m, 35f	17m, 8f	12m, 7f
Age in days (SD)	447.74 (18.31)	449.14 (21.2)	444.76 (23.71)	451.21 (19.25)

### Coding Scheme

Infant’s manual motor behaviours were coded for as long as the blocks were in front of them. Broadly, behaviours fell into three categories: ‘reaches’, ‘hand movements’ and ‘object manipulation’. A motor behaviour was considered a reach if infants extended their hand to grasp an object. If, however, the infant extended their arm and placed their hand on top of the object, without the grasping motion, this was considered a ‘hand movement’. Other ‘hand movements’ included sub categories such as: waving hand/arm, placing hand in mouth, and sliding/swiping hand where the infant is moving their hand in the space around them without grasping or contacting a block. Any instance of the infant acting upon the block was considered ‘object manipulation’, e.g., banging blocks, throwing blocks, putting blocks in the mouth. Every behaviour was coded; for example, if the infant banged a block three times in a row, this would be considered three distinct ‘object manipulation’ behaviours. Every behaviour was also coded for which hand (left or right) carried it out. If both hands worked in unison, with actions starting and ending at the same time (e.g., hand waving where both hands synchronously/symmetrically carried out the action), this was coded as ‘both hands’ for the one action. If, however, the hands were not working in unison (but were engaging in similar actions asynchronously), this was considered to be two distinct actions and the handedness was assigned to each action separately. Crucially, every behaviour was also coded for whether infant’s crossed their body midline to engage in the behaviour. For example, did the right hand pass into the left side of space when the infant was executing a reach? If the hand crossed the body midline, this was considered a ‘contralateral’ action. If the hand did not pass the body midline and stayed within the same side of space, this was considered an ‘ipsilateral’ action. Every action was also marked with the time stamp in the video for when the behaviour occurred.

All videos were coded by one researcher (JBA). Twelve percent of the videos were randomly selected and double coded by a second researcher who was blind to the group status of each infant. Intraclass correlations (two way mixed; single measures; absolute agreement) were conducted on the coded variables. A high level of reliability was found for the following coded behaviours: ‘Total midline crossings’ (ICC = 0.86,) ‘Contralateral hand movements’ (ICC = 0.73), ‘Contralateral object manipulations’ (ICC = 0.79), ‘Ipsilateral reaches’ (ICC = . 84), ‘Ipsilateral object manipulations’ (ICC = 0.89) and ‘Total behaviours’ (ICC = .92). A moderate level of reliability was found for the following coded behaviours: ‘Contralateral reaches’ (ICC = 0.7) and ‘Ipsilateral hand movements’ (ICC = 0.67); all ps ≤ 0.001).

### Measures of ASD and ADHD Traits

As we were also interested in determining how related midline crossing behaviours were to phenotypical characteristics of ASD and/or ADHD, we also included data from a number of parent report measures at 2 years. At this age, we do not make clinical determinations about the presence or absence of ASD/ADHD. We are currently re-assessing this sample at an older age, and when that process is complete we will be able to show how the motor behaviours reported here relate to categorical diagnostic outcome.

Parents completed the Quantatitive Checklist for Autism in Toddlers (QChat; Allison et al. 2008) and the Child Behaviour Checklist (CBCL; Achenbach and Rescorla 2000, 2001) at the 24-month time point. These measures allow us to determine whether or not the infant motor behaviours reported associate with dimensional variation in later clinical traits. The QChat measures autistic traits in young toddlers in terms of domains relating to pretend play, joint attention, language development, repetitive behaviours and social communication. The questionnaire is comprised of 25 questions on a 5–6 point scale. Item scores are then summed to produce a total score. In regards to the CBCL, we were particularly interested in the ADHD sub-scale. This scale measures children’s inattentive and hyperactive behaviour for individuals aged 1.5–5 years of age. Parents are asked to indicate how well each statement describes their child’s behaviour as observed within the past 2 months on a 3-point Likert rating. Item scores are then summed to produce a total score. The CBCL manual recommends that missing items are treated as 0 (“Not True”); this approach is used here where at least 1 item rating on the ADHD scale is provided.

The CBCL has been used in a number of studies in the toddler and early childhood age range (e.g., O’Shea et al. 2014; Gagne et al. 2020). There is some evidence regarding its stability and predictive validity beyond toddlerhood, with research indicating that it maps well onto diagnostic criteria for ADHD in the DSM-5 (e.g., de la Osa et al. 2016; Kim and Ha 2019). For the purpose of this study, we are not using the CBCL as a means of ASD/ADHD diagnosis, but rather as a measure of ASD/ADHD related traits.

Further, trained researchers in the BASIS/STAARS teams administered the Autism Diagnostic Observation Schedule Toddler Module (ADOS-T; Luyster et al. 2009) at the 24-month time point. This measure is a semi-structured play based behavioural assessment that measures ASD traits.

### Mullen Scales of Early Learning (MSEL)

The MSEL (Mullen 1995) was administered at all time points by trained researchers in the STAARS team. To allow for the greatest level of replicability and consistency across examiners, we have extremely strict guidelines about how the MSEL should be administered and marked. To this end, our guidelines for Mullen scoring include only behaviours that are captured on camera (so can be confirmed by a second/third researcher if necessary) within the Mullen session. For example, if an infant demonstrates babbling throughout the rest of the testing day (i.e., during another task or a lunch break), but not during the specific Mullen administration session, we would not score this infant as being able to produce babbling sounds on the Expressive Language scale. To further ensure the fidelity of the scoring, a second fully trained researcher watches the administration in real time (via a video feed) and consensus discussions take place after the testing session. These strict administration and scoring guidelines (although those recommended in the Mullen manual) may not be those applied more broadly in the field, and thus may account for relatively poorer performance in this cohort at infant timepoints relative to US norms.

## Results

As there were very slight variations in the duration of the task (often due to ending the task earlier than 3 min because of infant fussiness), we calculated the “rate per minute” for each behaviour (frequency of the behaviour/duration of task).

Separate linear mixed models (LMMs) with the following dependent variables were run: ‘Total contralateral behaviours’, ‘Contralateral reaches’, ‘Contralateral hand movements’, ‘Contralateral object manipulations’, ‘Total ipsilateral behaviours’, ‘Ipsilateral reaches’, ‘Ipsilateral hand movements’, ‘Ipsilateral object manipulations’ and ‘Total behaviours’. We decided to decompose the ‘Total behaviours’ variable (for both contralateral and ipsilateral behaviours which are the sum of all three *types* of behaviour; reaches, hand movements and object manipulations), to examine if there was a type of motor behaviour that especially contributed to ipsilateral or contralateral behaviours.

All LMMs used the following fixed factors: Time point (5 months, 10 months, 14 months), Sex (male, female) and Likelihood (ASD-L, ADHD-L). The Likelihood factor was computed as follows: each infant was given a score of ‘0’ or ‘1’ for ASD and a score of ‘0’ or ‘1’ for ADHD, depending on familial history of these disorders. As such, an infant with a first degree relative with ASD, but not ADHD would be assigned a ‘1’ for ASD-L and a ‘0’ for ADHD-L. An infant with a first degree relative with ADHD, but not ASD would be assigned a ‘0’ for ASD-L and a ‘1’ for ADHD-L. An infant with familial history of both disorders would be assigned ‘1’ for ASD-L and ‘1’ for ADHD-L and an infant with no familial history of either disorder would be assigned a ‘0’ for ASD-L and a ‘0’ for ADHD-L. We computed likelihood in this way so we could examine the interaction of the two disorders and if there were any additive/protective effects of having an elevated likelihood of both disorders.

To account for slight variations in age across infants, infants’ exact age (in days) was added as a covariate to each model. The repeated covariance type was set as ‘compound symmetry’ and the maximum likelihood estimate was used for each model.

For follow up analyses (where LMMs indicated interactions between elevated likelihood groups) and figures, the more traditional elevated likelihood approach was taken and infants were split into four groups: EL-ASD, EL-ADHD, EL-ASD + ADHD and TL.

### Total Contralateral Behaviours

**Fig. 2:**
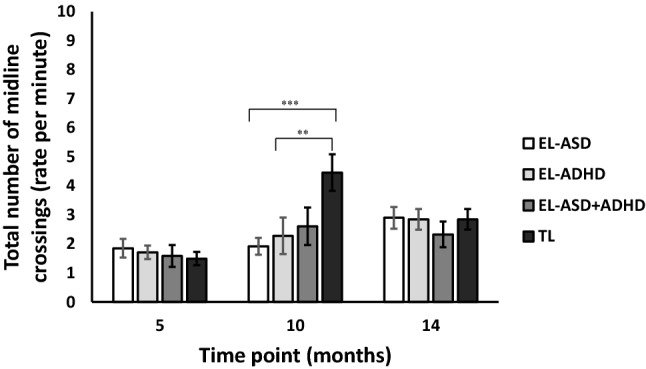
Graph showing the total number of contralateral behaviours across likelihood and age group

The LMM revealed a main effect of Time [F(2, 276) = 8.41, p < 0.001, *η*_*p*_^2^ = 0.06], with means indicating that the frequency of crossing the body midline to conduct a manual action in the contralateral side of space increased with age (see Fig. 2). Furthermore, the model showed a significant interaction of Time*ASD-L [F(2, 276) = 3.49, p = 0.032, *η*_*p*_^2^ = 0.03] and of Time*ASD-L*ADHD-L [F(2, 276) = 5.17, p = 0.006, *η*_*p*_^2^ = 0.04]. No other significant effects were found.

To examine the contributions of ASD and ADHD likelihood on contralateral behaviours, we conducted separate LMMs at each time point (using the same model parameters as above but removing Time point as a repeated and fixed factor). These showed there were no interaction effects of ASD-L*ADHD-L at 5 [F(1, 96) = 0.11, p = 0.74, *η*_*p*_^2^ = 0.001] or 14 months [F(1, 133) = 0.12, p = 0.73, *η*_*p*_^2^ = 0.001]. However, there was a significant interaction at 10 months [F(1, 146) = 10.54, p = 001, *η*_*p*_^2^ = 0.07]. Follow-up analyses showed that, at 10 months of age, the TL group engaged in more contralateral behaviours that involved crossing the midline than both the EL-ASD [t(100) = 5, p < 0.001, d = 1.15] and the EL-ADHD groups [t(49) = 3, p = 0.004, d = 0.9] and the EL-ASD + ADHD group, though the latter did not survive Bonferroni correction [t(43) = 2.15, p = 0.037, d = 0.62]. Further, no differences were found when comparing the EL-ASD group with EL-ADHD [t(99) = 1.1, p = 0.3, d = 0.21], EL-ASD with EL-ASD + ADHD [t(93) = 1.1, p = 0.26, d = 0.35] and the EL-ADHD group compared with the EL-ASD + ADHD group [t(42) = 0.25, p = 0.8, d = 0.16] at 10 months of age.

Following this set of analyses, we decomposed the Total number of contralateral behaviours into three categories: contralateral reaches, hand movements and object manipulations; the analyses for these sub categories of behaviours are presented below.

#### Contralateral Reaches

**Fig. 3:**
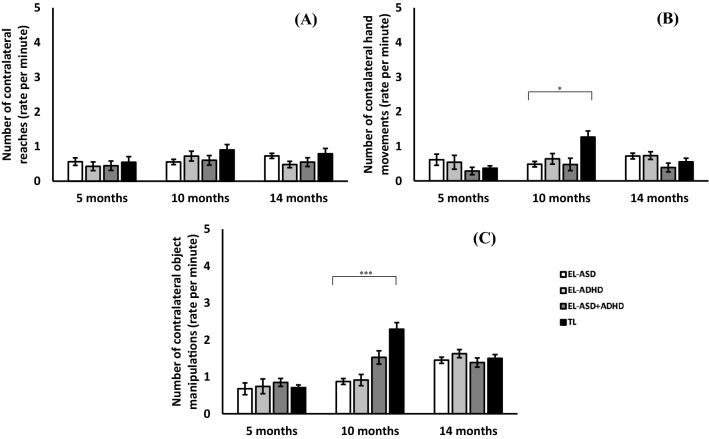
Graph showing the number of contralateral behaviours across likelihood and age groups. **a** Contralateral reaches, **b** contralateral hand movements and **c** contralateral object manipulations

The LMM analysis revealed a significant main effect of ADHD-L; [F(1, 161) = 4.64, p = 0.03, *η*_*p*_^2^ = 0.03], with means indicating that this group produced overall fewer reaches that involved crossing the midline (see Fig. 3a).

#### Contralateral Hand Movements

The LMM analysis revealed a significant main effect of ASD-L [F(1, 154) = 4.4, p = 0.038, *η*_*p*_^2^ = 0.03], with means indicating that those with a Typical Likelihood of ASD performed a greater number of contralateral hand movements compared to those with an elevated likelihood of ASD (see Fig. 3b). Further, a significant interaction effect of ASD-L*Time [F(2, 275) = 3.17, p = 0.044, *η*_*p*_^2^ = 0.02] was also found. Examining this interaction with pairwise comparisons, it was found that infants with a Typical Likelihood of ASD (TL) performed a greater number of contralateral hand movements compared to those with an elevated likelihood of ASD (EL-ASD) at the 10 month time point [t(100) = 4.59, p < 0.001, d = 1.05] but not at the 5 [t(68) = 1.07, p = 0.29, d = 0.27] or 14 [t(89) = 1.05, p = 0.3, d = 0.26] time points.

#### Contralateral Object Manipulations

The LMM revealed a significant main effect of Time [F(2, 268) = 6.56, p = 0.002, *η*_*p*_^2^ = 0.05] and Sex [F(1, 161) = 6.31, p = 0.013, *η*_*p*_^2^ = 0.04], with means indicating that there is an increase in these behaviours as infants age and that male infants carry out a greater number of object manipulations respectively.

Further to this, the analysis showed a significant interaction between Time*ASD-L*ADHD-L [F(2, 268) = 3.78, p = 0.024, *η*_*p*_^2^ = 0.03]. Examining the data at each time point, LMMs indicated that the above interaction was only significant at the 10 month time point [F(1, 146) = 10.54, p = 0.001, *η*_*p*_^2^ = 0.07] and not the 5 [F(1, 96) = 0.05, p = 0.83, *η*_*p*_^2^ = 0.001] or 14 month time points [F(1, 133) = 0.04, p = 0.84, *η*_*p*_^2^ = 0.0].

As such, follow up independent samples t-tests showed that the TL group crossed the midline to manipulate an object more than the EL-ASD group [t(100) = 3.88, p < 0.001, d = 0.89] (see Fig. 3c) and the EL-ADHD group, though the latter did not survive correction [t(50) = 2.4, p = 0.02, d = 0.68) and t(42) = 1.06, p = 0.3, d = 0.33, respectively]. The EL-ASD group showed fewer crosses than the EL-ASD + ADHD group, which again did not survive correction [t(92) = 2.16, p = 0.03, d = 0.57]. No differences were found when comparing EL-ASD with EL-ADHD [t(100) = 0.17, p = 0.87, d = 0.04], and EL-ADHD with the ASD + ADHD-L group [t(42) = 1.48, p = 0.15, d = 0.47].

### Total Ipsilateral Behaviours

**Fig. 4:**
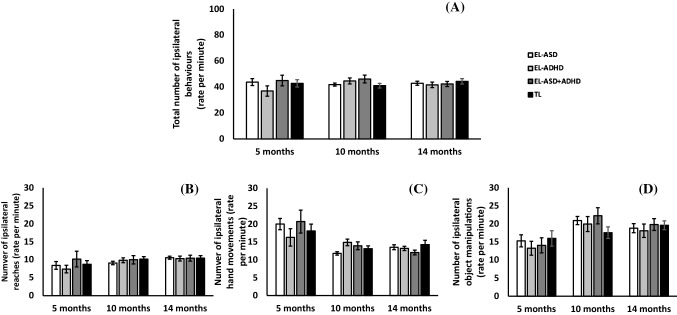
Graph showing the number of ipsilateral behaviours across likelihood and age group. **a** The total number of ipsilateral behaviours, **b** the number of ipsilateral reaches, **c** the number of ipsilateral hand movements and **d** the number of ipsilateral object manipulations

Control analyses of ipsilateral behaviours across Time (Fig. 4) indicated that effects were not due to performance of different numbers of behaviours overall (which could reduce the opportunities for contralateral manual behaviours). A LMM showed no main effect of Time [F(2, 272) = 0.46, p = 0.64, *η*_*p*_^2^ = 0.005], ASD-L [F(1,163) = 0.3, p = 0.59, *η*_*p*_^2^ = 0.004], ADHD-L [F(1, 163) = 0.11, p = 0.74, *η*_*p*_^2^ = 0.0] and no interaction of ASD-L*ADHD-L [F(1, 163) = 0.21, p = 0.65, *η*_*p*_^2^ = 0.004]. We also examined ipsilateral behaviours by category.

#### Ipsilateral Reaches

Ipsilateral reaches increased with Time [F(2, 274) = 4.2, p = 0.016, *η*_*p*_^2^ = 0.03] (see Fig. 4b). Additionally, there was a significant interaction of ADHD-L*Sex [F(1, 169) = 4.24, p = 0.041, *η*_*p*_^2^ = 0.02], with pairwise comparisons indicating that male infants in the EL-ADHD group performed a greater number of ipsilateral reaches than female infants in this group [t(28) = 2.07, p = 0.048, d = 0.8].

#### Ipsilateral Hand Movements

Ipsilateral hand movements increased with Time [F(2, 269) = 18.12, p < 0.001, *η*_*p*_^2^ = 0.12], but was not influenced by ASD-L [F(1, 152) = 0.08, p = 0.78, *η*_*p*_^2^ = 0.0], ADHD-L [F(1, 152) = 0.03, p = 0.87, *η*_*p*_^2^ = 0.0] or ASD-L*ADHD-L [F(1, 152) = 0.02, p = 0.89, *η*_*p*_^2^ = 0.0], (see Fig. 4c).

#### Ipsilateral Object Manipulations

Ipsilateral manipulations increased with Time [F(2, 270) = 8.8, p < 0.001, *η*_*p*_^2^ = 0.06], but did not interact with ASD-L [F(1, 157) = 0.7, p = 0.41, *η*_*p*_^2^ = 0.004], ADHD-L [F(1, 157) = 0.03, p = 0.87, *η*_*p*_^2^ = 0.0] or ASD-L*ADHD-L [F(1, 157) = 0.003, p = 0.95, *η*_*p*_^2^ = 0.0], (see Fig. 4d).

### Total Behaviours

**Fig. 5:**
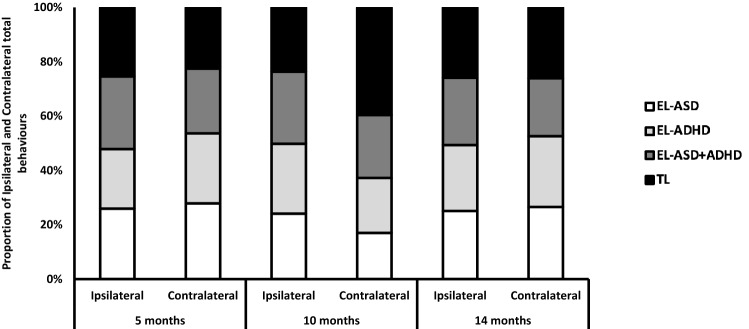
Graph showing the proportion of contralateral and ipsilateral behaviours across likelihood and age groups. **a** The contralateral and ipsilateral behaviours across all types of behaviour, **b** the proportion of contralateral and ipsilateral reaches, **c** the proportion of contralateral and ipsilateral hand movements and **d** the proportion of contralateral and ipsilateral object manipulations

The LMM showed a main effect of Sex [F(1, 163) = 4.1, p = 0.045, *η*_*p*_^2^ = 0.25] with means indicating that male infants perform more manual behaviours compared to female infants. Figure 5 shows the proportion of ipsilateral vs contralateral behaviours performed across likelihood groups and time points).

## General Motor Skills

An explanation for the group differences in manual contralateral behaviours could be that of a more generic motor delay in the Elevated Likelihood groups. As such, we examined the motor scales on the Mullen Scales of Early Learning (Mullen 1995). As mentioned in the ‘Methods’ section, we used strict administration and scoring guidelines for this behavioural assessment. Whilst, the TL group in this study may report slightly poorer performance relative to US norms, it is worth noting that there is stable individual variation in our Mullen scores across age points indicating internal consistency and that within-cohort comparisons are valid (see Jones et al. 2014), Further, at 2 years of age, participants are within the typical range of scores (see Table 3), which would suggest this group are typically developing.

Considering that we were only investigating this at the 10 month time point (where significant differences between groups had been shown previously), there was no need to conduct LMMs as above (although see Supplementary Materials for the 5 and 14 month time points). Instead, we conducted post-hoc analyses only. Independent samples t-tests demonstrated that there were no significant differences between the TL and EL-ASD groups on the Gross [t(101) = 1.46, p = 0.15, d = 0.33] or Fine motor scales [t(101) = 0.52, p = 0.61, d = 0.12] respectively (see Table 3 for Means and SE for all Mullen scales across all groups and time points). This pattern of findings was also true when comparing the TL group with the EL-ADHD and the EL-ASD + ADHD groups; no significant differences on the Gross [t(52) = 1.1, p = 0.27, d = 0.34; t(44) = 0.33, p = 0.7, d = 0.12] or Fine motor scales [t(52) = 0.06, p = 0.95, d = 0.01; t(44) = 0.63, p = 0.53, d = 0.19] respectively.

**Table 3:** Mullen scales of early learning *T* scores (means and SE) across likelihood and age groups

	TL	EL-ASD	EL-ADHD	EL-ASD + ADHD
5 months				
Fine motor	42.92 (2.05)	43.02 (1.34)	44.56 (1.57)	42.92 (2.27)
Gross motor	43.69 (1.85)	47.08 (1.09)	48.56 (1.7)	49 (1.17)
Visual reception	47.27 (1.24)	46.08 (1.35)	44.56 (1.97)	48.25 (2.52)
Expressive language	41.85 (1.46)	40.65 (1.05)	37.69 (2.75)	40.5 (2.15)
Receptive language	36.88 (2.21)	34.67 (1.72)	41.19 (3.51)	43.42 (4.21)
Composite score	85 (1.83)	82.96 (1.51)	84.5 (2.45)	87.75 (2.8)
10 months				
Fine motor	51.63 (2.48)	50.24 (1.34)	51.85 (2.64)	49.21 (2.93)
Gross motor	34.89 (2.27)	38.25 (1.11)	38.3 (2.05)	36.16 (2.2)
Visual reception	48.85 (1.54)	49.82 (1.08)	47.26 (1.86)	47.68 (1.77)
Expressive language	36.85 (1.9)	36.53 (1.47)	33.85 (2.34)	36.58 (3.42)
Receptive language	39.26 (1.72)	38 (1.21)	34.67 (1.96)	35.63 (2.47)
Composite score	88.89 (2.35)	88.03 (1.73)	84.67 (2.97)	85.42 (3.86)
14 months				
Fine motor	49.65 (2.54)	48.22 (1.36)	47.21 (2.51)	43.89 (2.27)
Gross motor	36.74 (2.79)	46.64 (1.55)	45.5 (2.21)	45.58 (2.64)
Visual reception	35.09 (1.85)	37.67 (1)	36.33 (1.19)	33.47 (1.49)
Expressive language	37.09 (1.83)	36.67 (1.33)	40.75 (1.91)	34.37 (3.15)
Receptive language	32.87 (1.35)	31.48 (1)	31.54 (1.88)	28.52 (2.45)
Composite score	78.78 (2.5)	78.37 (1.39)	79.08 (2.27)	72.53 (3.33)
24 months				
Fine motor	55.31 (2.49)	50.81 (1.32)	51.31 (2.28)	51.37 (2.76)
Visual reception	59.63 (2.18)	48.89 (1.62)	56.23 (2.55)	47.94 (2.57)
Expressive language	55.42 (2.51)	49.74 (1.86)	52.59 (2.93)	44.94 (2.77)
Receptive language	57.67 (1.78)	51.06 (1.73)	52 (2.95)	49 (2.6)
Composite score	114.25 (3.66)	100.63 (2.62)	106.86 (4.52)	96.94 (4.28)

Further, we also conducted a number of linear regression analyses to determine whether total midline crossings at 10 months predicted Gross and Fine motor skills at the 10 and 24 month time points (see Table 3). We found a marginally significant positively associative relationship between total midline crossings and Fine motor skills at 10 months [R^2^ = 0.03, F(1, 144) = 3.79, p = 0.054, *η*_*p*_^2^ = 0.03]. However, total midline crossings did not significantly predict Gross motor skills at the 10 month time point [R^2^ = 0.01, F(1, 144) = 1.6, p = 0.21, *η*_*p*_^2^ = 0.01] or Fine motor skills at 24 months of age [R^2^ = 0, F(1, 138) = 0.06, p = 0.81, *η*_*p*_^2^ = 0].

## Handedness

A further potential explanation could relate to infants’ handedness. To explore this, we first calculated the laterality quotient for each infant at the 10 month time point (proportion of ‘Right’ hand behaviours – proportion of ‘Left’ hand behaviours) and then used this quotient to classify hand dominance (negative values were considered to show a greater left hand dominance, whereas positive values indicated a greater dominance for the right hand). Considering that we were only investigating this at the 10 month time point (where significant differences between groups had been shown previously), there was no need to conduct LMMs as above. Instead, we conducted a mixed ANOVA to examine group differences in handedness. Here, we found a found a main effect of Handedness [F(1, 142) = 15.17, p < 0.001, *η*_*p*_^2^ = 0.1] with means indicating that participants performed more behaviours with their right hand compared to their left hand (indicating a right hand dominance across participants). However, there were no main or interaction effects of Group [F(3, 142) = 0, p = 1, *η*_*p*_^2^ = 0; F(1, 143) = 0.08, p = 0.97, *η*_*p*_^2^ = 0.002, respectively]. Further, the total number of Right/Left hand behaviours did not correlate with the proportion of total behaviours that involved crossing the midline (r = 0.06, n = 149, p = 0.47 and r = 0.09, n = 149, p = 0.26 for the Right and Left hand behaviours, respectively).

To examine whether TL infants perform more contralateral behaviours with their dominant hand, an analysis contrasting the proportion of contralateral behaviours performed with the dominant hand across group was conducted. A univariate ANOVA found no significant differences in the proportion of contralateral behaviours performed with the dominant hand across Group [F(3, 137) = 2.36, p = 0.08, *η*_*p*_^2^ = 0.05].

## Relationship with ASD and ADHD Traits

**Fig. 6:**
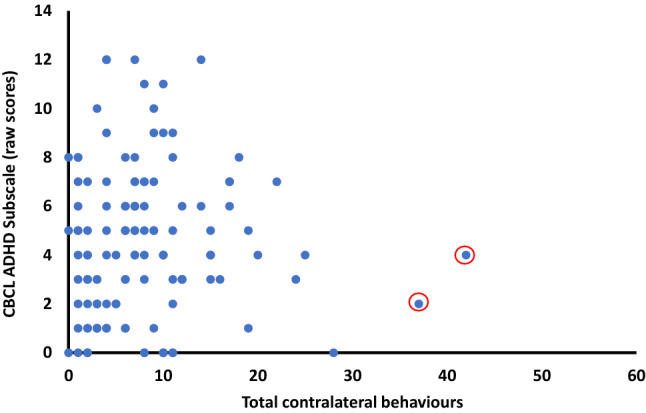
Graph showing the total number of contralateral behaviours at 10 months against ADHD subscale raw scores on the CBCL. Outliers outlined in red

We examined the relationship between the frequency of contralateral behaviours and whether they were related to ASD and/or ADHD traits. Examining scatter plots of total midline crosses by the various measures indicated 2 outliers (more than 4 standard deviations from the mean; see Fig. 6), which we removed. Then, we conducted a multiple regression analysis investigating the total midline crosses at 10 months of age with the raw scores from both the ADHD subscale of the CBCL (Achenbach and Rescorla 2000, 2001) and the QChat (Allison et al. 2008) at the 24 month time point in our EL groups (see Table 4). We found that total midline crosses performed at 10 months significantly associated with ADHD traits on the CBCL [R^2^ = 0.21, F(1, 91) = 4.26, p = 0.04, *η*_*p*_^2^ = 0.04], with more contralateral behaviours related to greater ADHD traits. However, total contralateral behaviours did not significantly predict ASD traits on the QChat [R^2^ = 0.14, F(1, 91) = 1.83, p = 0.18, *η*_*p*_^2^ = 0.02]. Including the entire EL sample and conducting nonparametric bivariate correlations, we found a significant relationship between total midline crossings and the CBCL ADHD subscale [r(94) = 0.21, p = 0.047] and a trend between total midline crossings and the QChat [r(94) = 0.16, p = 0.12].

**Table 4:** QChat, CBCL and ADOS-T consensus standard scores (means and SE) across typical and elevated likelihood cohorts

	TL	EL groups
CBCL (ADHD-subscale)	3.52 (0.49)	4.76 (0.32)
CBCL (PDP-subscale)	0.95 (0.31)	3.49 (0.45)
CBCL Total	19.23 (2.8)	35.8 (2.9)
QChat	46.77 (1.03)	47.75 (0.92)
ADOS-T (CSS Total)	1.63 (0.15)	2.94 (0.21)
ADOS-T (CSS Restrictive and Repetitive Behaviours)	2.79 (0.45)	3.59 (0.25)
ADOS-T (CSS Social affect)	1.63 (0.15)	3.38 (0.2)

To investigate whether midline crossing behaviours show specificity to one neurodevelopmental disorder (e.g., ADHD as suggested by the analysis above), we also examined the Pervasive Developmental Problems (PDP) subscale of the CBCL as well as the Total CBCL score. The PDP maps well onto DSM-5 criteria for ASD, whilst the Total CBCL score encompasses a range of internalising/externalising problem behaviours in children. A linear regression found no relationship between total contralateral behaviours and raw scores on PDP subscale [R^2^ = 0.005, F(1, 90) = 0.44, p = 0.51, η_*p*_^2^ = 0.005]. In order to examine the relationship between total midline crossings and the Total CBCL score, we conducted a multiple hierarchal regression analysis (enter method; so we could partial out ADHD subscale scores) and found that Total contralateral behaviours and ADHD CBCL scores explained a significant amount of variance in the CBCL Total score [F(2, 89) = 77.9, p < 0.001, η_*p*_^2^ = 0.63, R^2^ = 0.64, adj R^2^ = 0.63]. However, much of this explained variance was a result of the ADHD subscale score [Beta = 7.42, t(89) = 12.44, p < 0.001], with no relationship between total contralateral behaviours and CBCL Total scores [Beta = − 0.54, t(89) = − 1.31, p = 0.19]. Further to this, if we conduct the same multiple hierarchal regression analysis with the PDP subscale (and once again, the ADHD subscale partialled out), we find similar results; Total contralateral behaviours and ADHD CBCL scores explained a significant amount of variance in the CBCL PDP score [F(2, 90) = 30.97, p < 0.001, η_*p*_^2^ = 0.41, R^2^ = 0.41, adj R^2^ = 0.4]; however it is only the ADHD subscale that significantly explains the variance in the model [Beta = 0.94, t(90) = 7.82, p < 0.001].

The above measures were parent report questionnaires to examine ASD and ADHD traits. We also conducted a number of clinical/research based assessments to investigate the relationship between contralateral motor behaviours and ASD symptoms. For example, examining the ADOS-T, we found that total midline crosses performed at 10 months did not significantly predict ASD traits on the Calibrated Severity Score for Restrictive and Repetitive Behaviours [R^2^ = 0.01, F(1, 96) = 0.01, p = 0.91, *η*_*p*_^2^ = 0.0] or the Total Calibrated Severity [R^2^ = 0.15, F(1, 96) = 2.04, p = 0.16, *η*_*p*_^2^ = 0.02].

## Discussion

The current study was the first to examine midline crossing behaviours in a naturalistic, longitudinal design. We demonstrated, for the first time, that (at 10 months of age), infants with an Elevated Likelihood of ASD or ADHD produced fewer manual actions that involved their hand passing the body midline and crossing into the contralateral side of space. However, it must be noted this pattern of findings was only exhibited at the 10 month time point and not at either the 5 or 14 month time point. In comparison, our Typical Likelihood sample demonstrated ‘n shaped’ development, with midline crossing behaviours increasing significantly at 10 months (compared to the Elevated Likelihood groups) before decreasing at 14 months of age and becoming more in line with the EL groups. A number of control analyses ruled out differences in hand dominance and motor ability as possible explanations for this finding. Indeed, midline crossing did not seem to be related to large scale motor development. We discuss a number of potential accounts for our findings below.

Firstly, a possible suggestion is that our EL groups are more rigid in the way they segment peri-personal (near the body) space. It may be that infants with an Elevated Likelihood of ASD or ADHD segment space according to left/right parameters and are much less likely to cross the midline boundary with an ‘incongruent’ limb (e.g., the left hand acting in the right side of space and vice versa). Indeed, research with children with ASD has suggested that this population relies much more heavily on an anatomical reference frame when locating the hands in space (Wada et al. 2014); locating a limb is a necessary precursor to performing actions with that limb e.g., reaching. As such if, when locating a hand, individuals are more reliant of where it *usually* lies in space, perhaps they adopt more ‘usual’ postures and less ‘unusual’ postures, such as crossing over of the arm into the opposite side of space.

A second, related, explanation of our findings refers to attention shifting. Previous research has indicated that individuals with ASD have difficulties in disengaging from one stimulus and shifting attention to a second target (e.g., Landry and Bryson 2004; Richard and Lajiness-O’Neill 2015; Elsabbagh et al. 2009, 2013; see Landry and Parker 2013 for a meta-analysis of the gap-overlap literature in ASD populations), with further research indicating that they also demonstrate slower spatial visual orienting to visual stimuli (e.g., Sacrey et al. 2013; Townsend et al. 1996). Though less studied, similar things have been reported in older children and adolescents with ADHD (McAlonan et al. 2009). As such, it may be that those with an Elevated Likelihood of ASD and/or ADHD may initially attend to (and act within) the ipsilateral side of space. However, if they are slower (and less likely) to attend to the spatial array of visual targets that are in the contralateral side of space, it may also follow that they are less likely to act on these contralateral targets. We feel that this explanation is less compelling as we usually observe disengagement issues after 12 months of age in infants with an elevated likelihood of ASD (e.g., Elsabbagh et al. 2013; Sacrey et al. 2013).

A final explanation is that midline crossing behaviours may be related to multisensory integration abilities. We know that at 10 months, typically developing infants become more proficient in integrating visual, tactile and proprioceptive information and are able to update the location of their limbs when they are crossed over in space (Bremner et al. 2008; Rigato et al. 2014). It may be that the EL groups are less proficient in this integrative process and, as such, may engage in behaviours that require this process (e.g., midline crossing behaviours) much less than their TL counterparts. Indeed, multisensory integration has been identified as an area where ASD/ADHD populations demonstrate atypical patterns (e.g., Klin et al. 2009; Falck-Ytter et al. 2013, 2018; Panagiotidi et al. 2017; see Iarocci and McDonald 2006 for a review).

Further, it may be that these disrupted multisensory integration processes are also related to neural connectivity. Perhaps in typical development, at 10 months, there is an increase in both intra and inter-hemispheric connections in the brain (e.g., Thatcher et al. 2008; Xie et al. 2018), with particular emphasis on increased connections within the somatosensory network (see Nevalainen et al. 2014) and maturation of the corpus callosum (Ballesteros et al. 1993; Teicher et al. 2004; Barkovich and Kjos 1988). Increased neural connections within the somatosensory network and corpus callosum are required to initiate, or more efficiently, integrate inputs from vision, touch and proprioception which would allow infants to dynamically update where their limbs lie in space, allowing them to then act with these limbs. This increase in neural connections could also be a contributing factor to the range of motoric, limb movements and postures (e.g., crossing over themselves) the developing infant is capable of adopting. If these processes are disrupted in some way, and typical neural connectivity trajectories are not present (Piven et al. 1997; Vidal et al. 2006; Just et al. 2006; Alexander et al. 2007; Boger-Megiddo et al. 2006; Wolff et al. 2015; Haartsen et al. 2019) we may see reduced instances of infants engaging in midline crossing behaviours (as we do with our EL groups).

Related to the above explanation, Rigato et al. (2014) found a tentative relationship between midline crossing and tactile remapping. Here, it was found that 8 month old infants that engaged in at least one reach that involved crossing the midline, demonstrated tactile remapping abilities. In contrast, those infants that did not cross the midline when reaching did not show neural remapping of a tactile stimulus on the body. It may be that midline crossing abilities are related, in some way, to updating the posture of the limbs and somatosensory remapping. However, the current study is unable to tease apart this relationship. Future studies examining tactile remapping and midline crossing abilities in elevated likelihood groups are necessary to elucidate this relationship.

Other studies using the infant sibling design have found that infants with an elevated likelihood of ASD demonstrate delays in gross and fine motor behaviours such as reaching, object manipulation and sitting independently (Bhat et al. 2011; Koterba et al. 2014; Iverson and Wozniak 2007; Nickel et al. 2013; Focaroli et al. 2016; Libertus et al. 2014; Iverson et al. 2019; Ozonoff et al. 2008). As such, it may be that midline crossing behaviours is part of a broader range of early motoric differences within EL populations. However, it must be noted that we did not see a lot of the reported motor atypicalities in our sample (as measured by the MSEL gross and fine motor scales).

This is the first study to show early motor differences in infants with an elevated likelihood of ADHD. The findings suggest that these differences start early in life (within the first year) and demonstrate a very similar profile to that of ASD likelihood. Indeed, attention shifting and multisensory integration atypicalities have also been found in ADHD populations (e.g., Panagiotidi, et al. 2017; Perchet et al. 2001; McAlonan et al. 2009), thus the potential explanations outlined above hold for both EL groups. However, a point of note is that the majority of this previous research has been conducted on individuals (primarily adults) who already have a diagnosis of ADHD. What is especially novel about the current study is that early motor differences have been found in infants, in the first year of life, who have an increased likelihood of going on to develop ADHD, but who do not have a diagnosis of the disorder. The fact that these infants share the same patterns of behaviour as those with an elevated likelihood of ASD may point to a shared, generic difference in nervous system functioning across the two neurodevelopmental disorders. This is in line with research demonstrating that infants with an elevated likelihood of ADHD display higher levels of irritability and a reduced ability to self regulate (Sullivan et al. 2015; Gurevitz et al. 2014).

In terms of the TL group, we must ask what could explain the ‘n shaped’ developmental trajectory demonstrated in this group, with midline crossing behaviours peaking at 10 months (and significantly greater than the EL groups) and then decreasing dramatically to be more in line with the EL groups at 14 months? Here, we must reiterate that this was a fairly surprising finding given that previous research indicated incremental increases in midline crossing behaviours as typically developing infants aged (e.g., Van Hof et al. 2002). One possibility is that, initially, TL infants over engage in midline crossing behaviours. As infants age and learn about the arsenal of postures their limbs are capable of adopting, they begin to engage in postures that are *less effortful* and *more efficient* when executing a manual action (e.g., ipsilateral reaches). It is important to thus consider that EL groups may be engaging in more ‘efficient’ manual behaviours through completing primarily ipsilateral behaviours. When differences are found between EL groups and typical likelihood infants, researchers can often view these differences as challenges or difficulties for the EL groups. However, it is important to recognise that these differences may illustrate early strengths or compensatory mechanisms within the EL groups. This stresses the importance of examining neurodevelopmental disorders with the perspective of both strengths and limitations.

This leads us to the question of the relationship between midline crossing behaviours and ASD and/or ADHD traits. We found a predictive relationship between midline crossing and ADHD traits on the CBCL, suggesting that individuals who engage in more midline crossing behaviours also demonstrate more ADHD traits. Control analyses of the frequency of manual behaviours observed ruled this out as a possible explanation for our findings. If viewed in the context of the potential explanations we present above, one possibility for this relationship may be that ADHD traits may act as a protective factor against the effects of background ASD elevated likelihood. For example, perhaps ADHD traits reduce the rigidity in the way these infants segment peri-personal space or increase motor and/or perceptual nimbility, resulting in more behaviours that cross the body midline. To lend some support for this potential explanation, Gliga et al. (2018) examined visual foraging behaviours in infants with an older sibling with ASD and either low or high levels of hyperactivity and inattention. The researchers showed that, at 8 months of age, the EL-ASD low hyperactivity and inattention group were more likely to return to areas they had previously visited in the visual scene, as compared to the EL-ASD high hyperactivity and inattention group, which was more in line with results from the TL group. This study both demonstrates that ADHD traits mitigates EL-ASD behaviours and that ADHD traits may be linked to less rigid patterns of spatial exploration. However, these reduced levels of spatial rigidity may not be enough to completely overcome differences in midline crossing behaviours, which is why we see a significant difference between our EL groups with ADHD and the TL group.

Taken together, the present study found that infants with an Elevated Likelihood of ASD or ADHD (compared to infants with a Typical Likelihood of these disorders) demonstrated significantly fewer manual behaviours that involved the hand crossing the body midline and passing into the contralateral side of space. We have ruled out motor abilities and hand dominance as possible accounts of our findings. Importantly, we have found the same pattern of behaviour for two EL groups: EL-ASD and EL-ADHD, which points to subtle changes in motor behaviours that are not specific to either disorder. As such, midline crossing behaviours may potentially be a transdiagnostic early risk factor. This reiterates the necessity to examine behaviours across multiple disorders; as otherwise, we would have interpreted midline crossing behaviour differences to be syndrome specific to ASD or ADHD. Examining the relationship between this index and ASD/ADHD traits demonstrated a predictive relationship with ADHD traits; this would suggest that whilst midline crossing behaviour manifests in infancy in the same way in these EL cohorts (in that it is reduced), it may be more specifically related to ADHD traits later on in toddlerhood.

Furthermore, the current study employed a nuanced coding scheme that decomposed a broad behaviour (crossing the midline) into categories that tapped into subtly different types of behaviours. As a result of this nuanced coding scheme, one sub-category of crossing the midline behaviours (object manipulations) may lend itself to be a specific early risk marker, potentially differentiating between ASD and ADHD; however this requires further investigation.

## Electronic supplementary material

Below is the link to the electronic supplementary material.

Supplementary file1 (DOCX 711 kb)
