# Supplementary material for: Early Motor Differences in Infants at Elevated Likelihood of Autism Spectrum Disorder and/or Attention Deficit Hyperactivity Disorder
Source: J Autism Dev Disord. 2020 Apr 23;50(12):4367–84. doi: 10.1007/s10803-020-04489-1 (PMC7677154; doi:10.1007/s10803-020-04489-1)
Supplement: Supplementary file 1 — Supplementary file1 (DOCX 711 kb) [file 10803_2020_4489_MOESM1_ESM.docx]

Supplementary Materials

Participants

Information about diagnostic status was ascertained through a number of methods. Before families enrolled in the study, a telephone screening form was used to determine the presence of ASD and ADHD in family members. During their infant’s visit to the lab, the parent/caregiver also completed a “Medical and Psychiatric History Interview” (Appendix A) with the researcher. The telephone screening form and this formal interview at a study visit were the primary sources of information about diagnostic status. In addition, we asked for medical updates at each study visit and re-administered the Medical and Psychiatric History Interview at the 2-year timepoint. We also requested diagnostic letters and asked parents to complete the DAWBA (Goodman, Ford, Richards, Gatward & Meltzer, 2000) ASD and ADHD sections and these were reviewed by the senior clinician (TC). In addition, parents completed the Conners (Conners, 2008) (for ADHD) and the Social Communication Questionnaire (Rutter, Bailey & Lord, 2003) and Social Responsiveness Scale (Constantino, 2002; for ASD) on the family member with a diagnosis and where possible all other family members. This information is used to characterise our sample rather than for exclusionary purposes since, in the UK, NHS clinical diagnoses follow a gold-standard procedure including collation of information from parents, teachers and from in-person assessment that is beyond the scope of this study and more accurate than simple questionnaire measures.

Up to 30% of children with ASD meet criteria for ADHD when prospectively assessed (Simonoff, Pickles, Charman, Chandler, Loucas & Baird, 2008). In clinical practice, the prevalence of dual diagnosis is in practice much lower (Russell, Rodgers, Ukoumunne & Ford, 2014). Given the nature of the co-occurrence between ASD and ADHD and our longitudinal study, sometimes family members would have a suspected diagnosis of ADHD at study entry that would be confirmed later in the study; on other occasions, a family would enrol on the basis of an ASD diagnosis in an older sibling but by the end of the study, they would report that the same sibling was now undergoing assessment for suspected additional ADHD. Where possible, families who reported suspected ADHD at study entry were screened using a shortened version of the Conners. Families who screened positive on this instrument were then included as a confirmed case. However, it remains likely that within families with ASD, rates of actual ADHD are higher than those captured by our 1/0 diagnostically-based rating system. Families where there was significant diagnostic uncertainty about the presence of either ASD or ADHD including (those that were screened for ADHD; n=12) were removed in a sensitivity analysis to check whether results differed substantially.

Results

The analyses conducted were repeated, however with data from 12 infants omitted. This was due to the fact that the elevated likelihood category for these infants was less concrete (see Table S1 for participant characteristics for this sub-sample). As such, we decided to remove these infants from the analyses in order to determine if their exclusion changed any of our findings.

Total contralateral behaviours:

The LMM revealed a main effect of Time [F(2, 256) = 9.27, p <.001, *η_p_*^2^ = .07], with means indicating that the frequency of crossing the body midline to conduct a manual action in the contralateral side of space increased with age. Additionally, a main effect of Sex was found [F(1, 155) = 3.86, p = .05, *η_p_*^2^ = .02], with means indicating that male infants produced more contralateral behaviours than female infants. Furthermore, the model showed a significant three-way interaction of Time*ASD-L*ADHD-L [F(2, 256) = 6.07, p =.003, *η_p_*^2^ = .04].

To examine the contributions of ASD and ADHD likelihood on contralateral behaviours, we conducted separate LMMs at each time point (using the same model parameters above, but removing Time point as a repeated and fixed factor). These showed there were no interaction effects of ASD-L*ADHD-L at 5 [F(1, 81) = 0, p = 1, *η_p_*^2^ = 0] or 14 months [F(1, 117) = .2, p = .66, *η_p_*^2^ = .001]. However, once again there was a significant interaction at 10 months [F(1, 128) = 12.37, p = 001, *η_p_*^2^ = .09].

As such, follow up independent samples t-tests showed a significant difference between the TL and EL-ASD group [t(100) = 5, p <.001, d = 1.15] with the TL group engaging in more contralateral behaviours that involved crossing the midline. Additionally, the TL group also engaged in more contralateral behaviours when compared with the EL-ADHD group [t(43) = 2.84, p = .007, d = .88]. Further, when the contribution of EL-ASD+ADHD on the total contralateral behaviours was compared with the TL group, no significant differences were found [t(39) = 1.48, p = .15, d = .5].

Further, no differences were found when comparing EL-ASD with EL-ADHD [t(97) = 1, p = .33, d = .24], EL-ASD with EL-ASD +ADHD [t(90) = 1.66, p = .1, d = .5] and individuals with just EL-ADHD compared with those with EL-ASD +ADHD [t(39) = 1.48, p = .15, d = .49].

Contralateral reaches:

The model showed a significant main effect of Time [F(2, 260) = 3.44, p = .033, *η_p_*^2^ = .03] and an interaction of ADHD*Sex approached significance [F(2, 147) = 3.75, p = .055, *η_p_*^2^ = .05], with means indicating that male infants performed a greater number of contralateral reaches than female infants.

Contralateral hand movements

The model showed a significant three-way interaction of Time*ASD-L*ADHD-L [F(2, 258) = 4.75, p =.009, *η_p_*^2^ = .04]. Once again, to examine the contributions of ASD and ADHD likelihood on contralateral hand movements, we conducted separate LMMs at each time point. These showed there were no interaction effects of ASD-L*ADHD-L at 5 [F(1, 89) = 1.59, p = .21, *η_p_*^2^ = .02] or 14 months [F(1, 125) = 2.52, p = .12, *η_p_*^2^ = .02]. However, once again there was a significant interaction at 10 months [F(1, 136) = 5.71, p = .018, *η_p_*^2^ = .04].

As such, follow up independent samples t-tests (examining the 10-month time point) showed a significant difference between the TL and EL-ASD group [t(100) = 4.59, p <.001, d = 1.05] with the TL group engaging in more hand movements that involved crossing the midline. Additionally, the TL group also engaged in more contralateral hand movements when compared with the EL-ADHD group [t(43) = 2.77, p = .008, d = .86]. When the contribution of EL-ASD+ADHD on contralateral hand movements was compared with the TL group, this was also found to be significant [t(39) = 2.51, p = .017, d = .83], but did not survive Bonferroni correction.

No further differences were found when comparing EL-ASD with EL-ADHD [t(93) = .5, p = .62, d = .13], EL-ASD with EL-ASD+ADHD [t(89) = .34, p = .73, d = .1] and EL-ADHD compared with EL-ASD+ADHD [t(32) = .08, p = .94, d = .003].

Contralateral object manipulations

The LMM revealed a significant main effect of Time [F(2, 251) = 6.71, p = .001, *η_p_*^2^ = .05] and Sex [F(1, 153) = 7.22, p = .008, *η_p_*^2^ = .05], with means indicating that there is in increase in these behaviours as infants age and that male infants carry out a greater number of object manipulations respectively.

Further to this, the analysis showed a significant interaction between ASD-L*ADHD-L [F(1, 153) = 4.01, p = .047, *η_p_*^2^ = .03] and Time*ASD-L*ADHD-L [F(2, 251) = 5.01, p = .007, *η_p_*^2^ = .04]. Examining the data at each time point, LMMs indicated that the above interaction was only significant at the 10 month time point [F(1, 136) = 12.92, p < .001, *η_p_*^2^ = .09] and not the 5 [F(1, 89) = .81, p = .37, *η_p_*^2^ = .009] or 14 month time points [F(1, 125) = .06, p = .82, *η_p_*^2^ = 0].

As such, follow up independent samples t-tests, examining the 10-month time point, showed a significant difference between the TL and EL-ASD groups [t(100) = 3.88, p <.001, d = .89] with the TL group engaging in more object manipulations that involved crossing the midline. Additionally, the TL group produced more contralateral object manipulations compared to the EL-ADHD group, however this did not survive Bonferroni correction [t(43) = 2.19, p = .034, d = .68]. Additionally, no significant differences were found between EL-ASD+ADHD and the TL groups [t(39) = .7, p = .49, d = .23].

The EL-ASD group were found to engage in fewer contralateral object manipulations than the EL-ASD+ADHD group [t(89) = 2.67, p = .009, d = .76], although this did not survive Bonferroni correction. Further, no differences were found when comparing EL-ASD with EL-ADHD [t(93) = .11, p = .91, d = .03] and EL-ADHD with EL-ASD+ADHD [t(32) = 1.8 p = .08, d = .65].

Total ipsilateral behaviours

No significant effects.

Ipsilateral reaches

The LMM revealed a significant main effect of Time [F(2, 252) = 3.64, p = .028, *η_p_*^2^ = .03], with reaches increasing with age.

Ipsilateral hand movements

The LMM revealed a significant main effect of Time [F(2, 250) = 19.32, p <.001, *η_p_*^2^ = .13], with means indicating the number of ipsilateral hand movements increases as infants aged.

Ipsilateral object manipulations

The LMM revealed a significant main effect of Time [F(2, 248) = 9.03, p < .001, *η_p_*^2^ = .07], with means indicating this increases as infant’s age.

Total behaviours

No significant effects.

The above results largely mirror those presented in the main text, with the full sample of infants. As such, we have demonstrated that the inclusion of these 12 infants in the main analysis does not distort the results. Any further exploratory analyses conducted will include our full sample.

General motor skills

Analyses in the main text demonstrated that, at the 10 month time point, the four groups did not differ in terms of their gross and fine motor skills (as measured by the MSEL; Mullen, 1995). To examine if there were any differences between groups at 5 and 14 months of age, we conducted a LMM (as we had more than one time point). Here, for Fine Motor skills, we found a significant main effect of Time [F(1, 141) = 6.43, p = .012, *η_p_*^2^ = .04] but no group effects. When examining Gross Motor skills, we found significant main effects of Time [F(1, 134) = 5.7, p = .018, *η_p_*^2^ = .04] and ADHD-L [F(1, 154) = 4.39, p = .038, *η_p_*^2^ = .03] and a significant interaction of ASD-L*ADHD-L [F(1, 154) = 4.16, p = .043, *η_p_*^2^ = .03].

To scrutinise this significant interaction further, we conducted independent samples t-tests, comparing the different likelihood groups on Gross Motor skills. As there were no interaction effects of Time, we collapsed the Gross Motor scores from the 5 and 14 month time points. We found a significant difference between the TL and EL-ASD groups [t(102) = 2.44, p = .016, d = .54] and the TL and EL-ADHD groups [t(56) = 2.23, p = .03, d = .6]; with the TL group demonstrating poorer gross motor skills than the elevated likelihood groups,

although this latter test did not survive Bonferroni correction (p = .017). We found no significant difference between the TL and EL-ASD+ADHD groups [t(45) = 1.92, p = .06, d = .58].

Mullen motor scales

Analyses in the main text demonstrated that manual contralateral behaviours was not related to gross and fine motor abilities as measured by the Mullen Scales of Early Learning (Mullen, 1995). In a secondary analysis, we controlled for overall motor expertise by including infant’s Fine and Gross motor raw scores as a covariate in our LMMs (at the 10 month time point specifically). We had decided not to do this as part of our primary analytical plan as the direction of causality may be less clear (i.e., it may be that aspects of motor behaviour that underlie reduced instances of contralateral behaviours also contribute to poorer performance on the Gross and Fine motor scales of the MSEL). All LMMs used the following fixed factors: Sex (male, female) and Likelihood (ASD-L, ADHD-L). Covariates in the LMMs included: age in days, Mullen Fine motor raw score and Mullen Gross motor raw score. The repeated covariance type was set as ‘compound symmetry’ and the maximum likelihood estimate was used for each model.

Total contralateral behaviours

We found significant effects of ASD-L [F(1, 145) = 7.95, p = .005, *η_p_*^2^ = .05], ADHD-L [F(1, 145) = 3.85, p = .05, *η_p_*^2^ = .03] and ASD-L*ADHD-L [F(1, 145) = 10.36, p = .002, *η_p_*^2^ = .07].

Contralateral reaches

We found significant effects of ASD-L [F(1, 145) = 5.21, p = .02, *η_p_*^2^ = .03] and ADHD*Sex [F(1, 145) = 5, p = .03, *η_p_*^2^ = .03].

Contralateral hand movements

We found significant effects of ASD-L [F(1, 145) = 11.12, p = .001, *η_p_*^2^ = .07], ADHD-L [F(1, 145) = 5.25, p = .02, *η_p_*^2^ = .03] and ASD-L*ADHD-L [F(1, 145) = 4.32, p = .04, *η_p_*^2^ = .03].

Contralateral object manipulations

We found a main significant effect of Sex [F(1, 145) = 5.77, p = .02, *η_p_*^2^ = .04] and a significant interaction of ASD-L*ADHD-L [F(1, 145) = 10.38, p = .002, *η_p_*^2^ = .07].

The above results largely mirror those presented in the main text, with motor development not covaried. As such, we have demonstrated that the exclusion of these variables in the main analysis does not distort the results.

Relationship with motor behaviours

Whilst analyses in the main text demonstrated that manual contralateral behaviours was not related to gross and fine motor abilities as measured by the Mullen Scales of Early Learning (Mullen, 1995), it could be argued that these scales are not tapping into the motor behaviours that may precede and/or facilitate midline crossing. A potential explanation for our findings may be that infants that had more motoric opportunities for crossing the midline may engage in this behaviour more. As such, we measured the correlation between the frequency of total contralateral behaviours in the observed task and parental reporting of how often infants engaged in specific motor behaviours in the 2 weeks before the lab session. The motor behaviours of particular interest were: sitting independently, crawling and cruising. Parents were asked if their infant had engaged in the behaviour “0 times, once, a few times or many times” over the last 2 weeks (see Figure S1). Researchers also rated how often they had observed the infant engaging in these behaviours over the course of the testing session. Of note is that (out of the entire sample), 6, 8 and 47 infants (across all likelihood groups) were not sitting independently, crawling or cruising respectively at the 10 month time point. Bivariate correlations found no relationship between total contralateral behaviours at 10 months of age with any of the motor behaviours as reported by the parent or the examiner (see Table S2).

We also examined the relationship between midline crossing and Visual Reception scores at the 10 and 24 month time points as visual reception often involves a motor component (e.g., head stability is necessary to be able to visually track a moving target, head turning to locate a sound and a number of the items on this scale require the infant to reach for things). Whilst midline crossing did not predict Visual Reception scores at 24 months [R^2^ = .003, F(1, 122) = .4, p = .81, *η_p_*^2^  = .003], it did at 10 months of age [R^2^ = .04, F(1, 144) = 5.15, p = .025, *η_p_*^2^  = .03]. We found this to be an inverse relationship, with more midline crossing behaviours related to poorer visual reception scores (see Figure S3).

To examine exactly what midline crossing behaviours may be tapping into, we investigated whether the change in midline crossing frequency (from 5 to 10 months and 10 to 14 months) predicted developmental change in the same time span (as measured by the MSEL and the parent reported Vineland Adaptive Behaviour Scale; VABS, Sparrow Cicchetti, Balla & Doll, 2005) in our TL sample. We calculated a per day rate of change for each variable (change in raw e.g., motor scores/change in age in days). These linear regressions showed no significant predictive relationships, all ps > .08 (see Tables S3.1 and 3.2).

Tables and Figures

Table S1

|  | **TD** | **ASD-L** | **ADHD-L** | **ASD+ADHD-L** |
| --- | --- | --- | --- | --- |
| **5 months** | | | | |
| *n* | 26 | 52 | 11 | 10 |
| Gender | 17m, 9f | 26m, 26f | 7m, 4f | 6m, 4f |
| Age in days (SD) | 177.42 (13.69) | 174.65 (20.18) | 166.73 (15.42) | 178.5 (14.25) |
| **10 months** | | | | |
| *n* | 27 | 77 | 20 | 16 |
| Gender | 16m, 11f | 39m, 38f | 12m, 8f | 9m, 7f |
| Age in days (SD) | 321.93 (16.7) | 319.53 (14.84) | 324.2 (30.77) | 319.38 (15.78) |
| **14 months** | | | | |
| *n* | 23 | 73 | 20 | 15 |
| Gender | 13m, 10f | 38m, 35f | 14m, 6f | 9m, 6f |
| Age in days (SD) | 447.74 (18.31) | 449.14 (21.2) | 445.3 (24.3) | 452.27 (21.05) |

Table S2

|  | **Sitting frequency (parent report)** | **Crawling frequency (parent report)** | **Cruising frequency (parent report)** | **Sitting frequency (examiner report)** | **Crawling frequency (examiner report)** | **Cruising frequency (examiner report)** |
| --- | --- | --- | --- | --- | --- | --- |
| **Total contralateral behaviours** |  |  |  |  |  |  |
| **Spearman’s rho correlation** | .12 | .05 | .16 | .13 | .02 | .09 |
| **Sig.** | .15 | .6 | .08 | .15 | .88 | .33 |
| **n** | 139 | 140 | 127 | 125 | 120 | 109 |

Table S3.1

| **Dependent variable** | **Age points** | **R^2^** | **F** | **p** |
| --- | --- | --- | --- | --- |
| **Fine motor** | 5-10 months | .15 | 3.39 | .08 |
|  | 10-14 months | .04 | .62 | .44 |
| **Gross motor** | 5-10 months | 0 | .008 | .93 |
|  | 10-14 months | .01 | .09 | .76 |
| **Visual reception** | 5-10 months | 0 | .006 | .94 |
|  | 10-14 months | .03 | .57 | .46 |
| **Receptive language** | 5-10 months | .12 | 2.63 | .12 |
|  | 10-14 months | .004 | .07 | .79 |
| **Expressive language** | 5-10 months | .008 | .15 | .71 |
|  | 10-14 months | .03 | .48 | .5 |

Table S3.2

| **Dependent variable** | **Age points** | **R^2^** | **F** | **p** |
| --- | --- | --- | --- | --- |
| **Motor** | 5-10 months | .1 | 1.44 | .25 |
|  | 10-14 months | .14 | 2 | .19 |
| **Language** | 5-10 months | .01 | .14 | .72 |
|  | 10-14 months | .001 | .008 | .93 |
| **Caring for self** | 5-10 months | .07 | 1.12 | .31 |
|  | 10-14 months | .13 | 1.84 | .2 |

Figure S1

Figure S2

Figure S3

Table headings

Table S1: Participant characteristics of a subset of participants included in the supplementary analyses, across likelihood and age groups

Table S2: Bivariate correlations examining the relationship between the total number of contralateral behaviours at 10 months and specific motor behaviours, as reported by parents and examiners

Table S3.1: Linear regressions with the change in contralateral behaviours predicting changes in each scale of the MSEL

Table S3.2: Linear regressions with the change in contralateral behaviours predicting changes in each scale of the VABS

Figure headings

Figure S1: Graph showing the proportion of infants who were sitting, crawling and cruising as reported by parents at the 10 month time point

Figure S2: Graph showing the proportion of infants’ proportion of total ipsilateral vs contralateral behaviours across frequency of sitting, crawling and cruising (across EL groups) as reported by parents at the 10 month time point

Figure S3: Scatter plot showing infants’ total contralateral behaviours and visual reception scores on the MSEL at 10 month time point

Appendix A
